# Human rather than ape-like orbital morphology allows much greater lateral visual field expansion with eye abduction

**DOI:** 10.1038/srep12437

**Published:** 2015-07-20

**Authors:** Eric Denion, Martin Hitier, Eric Levieil, Frédéric Mouriaux

**Affiliations:** 1 Inserm, U 1075 COMETE, Avenue de la côte de nacre, Caen, 5 Avenue de la côte de nacre, 14033 Caen cedex 9, France; 2 Department of Ophthalmology, CHU de Caen, Avenue de la côte de nacre, 14033 Caen cedex 9, France; 3 Medical School, Unicaen, pôle des formations des recherches en santé, 2 rue des Rochambelles, CS 14032, 14032 Caen cedex, France; 4 Department of Otolaryngology - Head & Neck Surgery CHU de Caen, Avenue de la côte de nacre, 14033 Caen cedex 9, France; 5 Anatomy Laboratory, pôle des formations des recherches en santé, 2 rue des Rochambelles, CS 14032, 14032 Caen cedex; 6 Cleverest Code, 24 place Etienne Pernet, 75015 Paris, France; 7 Department of Ophthalmology, CHU Pontchaillou, 2 rue Henri Le Guilloux, 35033 Rennes Cedex 9, France; 8 Université de Rennes 1, 2 rue du Thabor CS 46510, 35065 Rennes cedex, France

## Abstract

While convergent, the human orbit differs from that of non-human apes in that its lateral orbital margin is significantly more rearward. This rearward position does not obstruct the additional visual field gained through eye motion. This additional visual field is therefore considered to be wider in humans than in non-human apes. A mathematical model was designed to quantify this difference. The mathematical model is based on published computed tomography data in the human neuro-ocular plane (NOP) and on additional anatomical data from 100 human skulls and 120 non-human ape skulls (30 gibbons; 30 chimpanzees / bonobos; 30 orangutans; 30 gorillas). It is used to calculate temporal visual field eccentricity values in the NOP first in the primary position of gaze then for any eyeball rotation value in abduction up to 45° and any lateral orbital margin position between 85° and 115° relative to the sagittal plane. By varying the lateral orbital margin position, the human orbit can be made “non-human ape-like”. In the *Pan*-like orbit, the orbital margin position (98.7°) was closest to the human orbit (107.1°). This modest 8.4° difference resulted in a large 21.1° difference in maximum lateral visual field eccentricity with eyeball abduction (*Pan*-like: 115°; human: 136.1°).

The Hominoidea superfamily^1^ (“hominoids”) is comprised of modern humans (*Homo sapiens*) and non-human apes. Non-human apes, humans’ closest relatives^2^,^3^, include gibbons (family Hylobatidae), orangutans (family Hominidae, genus *Pongo*), chimpanzees and bonobos (family Hominidae, genus *Pan*) and gorillas (family Hominidae, genus *Gorilla*)^1^. Modern humans’ orbital morphology is unique among the Hominoidea superfamily in that the human orbital width/height ratio is highest and, whilst convergent (front-facing), the human orbit has the rearmost temporal orbital margin^4^,^5^. This orbital margin configuration increases the human median temporal visual field surface area by 46% with eye-abduction, which promotes effective visual and visual field exploration through eye motion rather than head motion^4^,^6^,^7^.

The neuro-ocular plane (NOP) is defined as the plane which, in the primary position of gaze (looking straight ahead in the distance), contains the centre of both crystalline lenses, optic discs, and optic foramina^8^,^9^,^10^. This plane can be used to obtain head orientations in space to facilitate precise comparisons between human and non-human apes^4^,^10^. In this plane, the human temporal orbital margin is 107.1°^4^,^5^ from the sagittal plane compared to 98.7°^4^,^5^ in humans’ closest relatives^3^,^11^ chimpanzees and bonobos. The visual field, including the additional visual field gained through eye motion, may be easily tested in humans^6^,^7^ but not in non-human apes. Visual field testing is not, therefore, an appropriate solution for appraising how the anatomical differences in the orbital margin between humans and non-human apes translate into visual field differences. To address this issue, we developed a mathematical model. The aim of the model was to calculate temporal visual field eccentricity in the NOP, first in the primary position of gaze (with eyes looking straight ahead), then for any eyeball rotation value in abduction up to 45° and for any temporal orbital margin position between 85° and 115°. In so doing, we aimed to quantify the influence of the orbital morphology difference between humans and non-human apes on temporal visual field extent, including the additional visual field gained through eyeball abduction.

## Results

### Neutral *φ* angle value

The neutral *φ* angle value is 94.9°. In our model, for an orbit with this *φ* value, eye abduction does not offer any additional visual field gain, taking the whole range of abduction into account.

### ω max., ω mean, ω top max. and ε max. results

**Figure 1:**
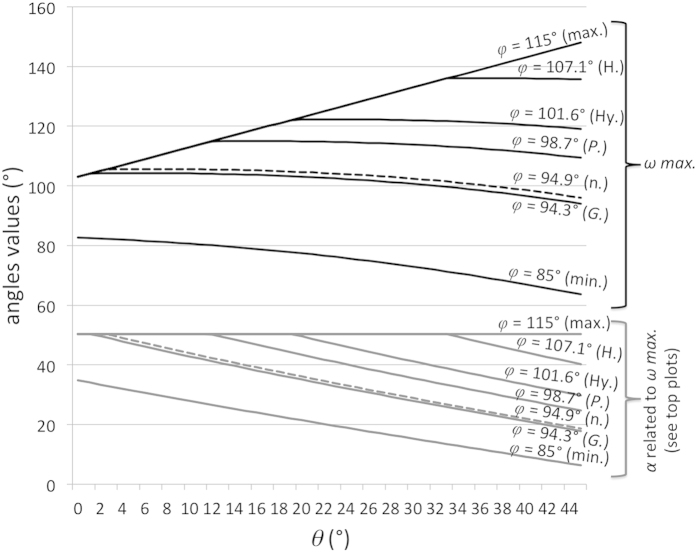
Plots of *ω max.* and related *α* angle according to varying *θ* angle values between 0° (primary position of gaze) and 45°. The plots of different *φ* (opening angle) are represented: maximum value recorded (max.) = 115°; Human (H.) = 107.1°; Hylobatidae-like (Hy.) = 101.6°; *Pan*-like (*P.*) = 98.7°; *Gorilla*-like (*G.*) = 94.3°; minimum value recorded (min.) = 85°. The neutral (n.) *φ* value (94.9°) is the one culminating in an *ω mean* value equal to that of *ε max.* taking the whole range of *θ* values (between 0 and 45°) into account. The *Pongo*-like orbit plot, the *φ* (94.4°) of which is very close to that of the *Gorilla*-like orbit (94.3°), has not been represented.

The *ω max.* (and related *α*) plots according to the various eye abduction values (*θ* from 0 to 45°) for different *φ* values are displayed in Fig. 1: The human orbit gives far more visual field expansion with eye abduction than modified non-human ape-like orbits. The *Gorilla*-like modified orbit plot is below that of the modified neutral orbit (in which the *φ* angle value is 94.9°). For each orbit type, the maximum *α* value decreases after the *ω top max.* value has been reached.

**Figure 2:**
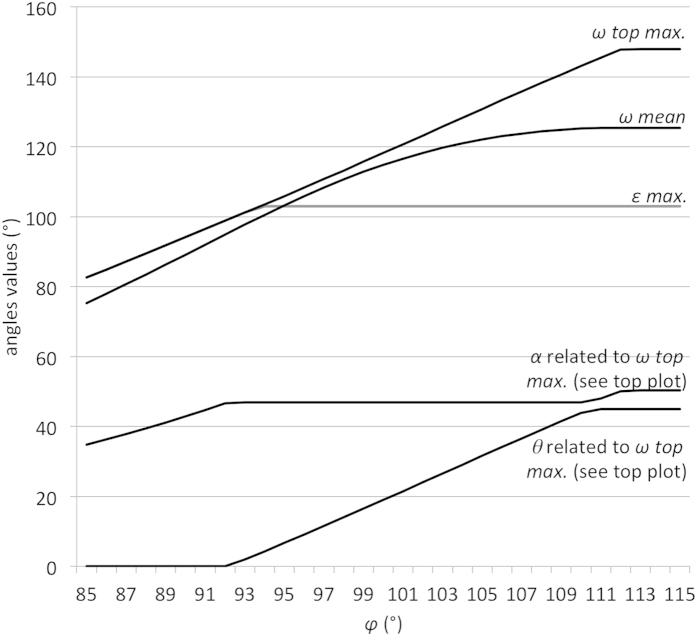
Plots of various angles for the human orbit according to varying eye abduction angles. Top section: plots of *ω top max.*, *ω mean, ε max.*, according to *φ* values between 85° (lowest recorded value) and 115° (highest recorded value). Bottom section: plots of *α* (up to 50.3°) and *θ* angle (from 0° to 45°) values related to *ω top max.* values.

Figure 2 displays the plots of *ω top max.* (and related *α* and *θ*), *ω mean*, *ε max.* according to *φ* angle values between 85° (lowest recorded value) and 115° (highest recorded value). The *ω mean* and *ε max.* plots intersect at 94.9° (neutral *φ* value). The *α* and θ values reach their respective maximum values (50.3° and 45°) with the highest *φ* values only.

**Figure 3:**
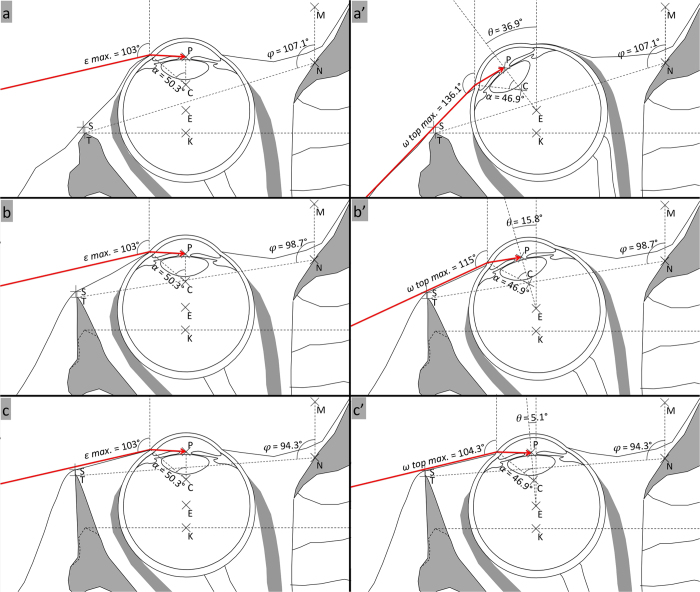
Schematic cross-sections illustrating *ε max.* and *ω top max.* values for the human orbit and two modified non-human ape-like orbits. Schematic cross-sections of the Human orbit (**a** and **a’**; *φ* = 107.1°), modified “*Pan*-like” orbit (**b** and **b’**; *φ* = 98.7°), and modified “*Gorilla*-like” orbit (**c** and **c’**; *φ* = 94.3°). In **a**, **b** and **c**, the eye is in primary position of gaze. The *ε max.* value is 103° for each orbit type. In **a’**, **b’**, **c’**, the eye is in the abduction position (denoted by *θ*) that yields the *ω top max.* value. In the human orbit, *ω top max.* is 136.1° for 36.9° abduction. In the modified “*Pan*-like” orbit, *ω top max.* is 115° for 15.8° abduction. In the modified “*Gorilla*-like” orbit, *ω top max.* is 104.3° for 5.1° abduction.

Schematic cross-sections illustrating *ε max.* and *ω top max.* values for the human orbit and modified *Pan*-like and *Gorilla*-like orbits are displayed in Fig. 3. For each orbit type, the visual field in the primary position (*ε max.*) extends 103°. With permitted eye-abduction, the visual field (*ω top max.*) extends 136.1° for the human orbit, 115° for the *Pan*-like orbit and 104.3° for the *Gorilla*-like orbit. This decrease in *ω top max.* values is accompanied by a decrease in related eye abduction (respective *θ* angles: 36.9°; 15.8°; 5.1°).

Information about the skulls included in this study and a summary of the main results generated by the mathematical model (*ε max.*, *ω top max.* with related *θ* and *α* angles; *ω mean* with related *θ* and *α* angles) are displayed in Table 1.

### Oculo-orbital indices (OOI) for modified (“non-human ape-like”) human orbits

The calculated OOIs in Hylobatidae, *Pan*, *Pongo* and *Gorilla* were 50.2%, 42%, 29.6% and 29.3%, respectively.

## Discussion

In 1961, Hedblom reported that humans could extend their visual field through eye motion, mostly in the temporal sector where the facial relief (brow, nose, cheek) does not interfere with vision^12^. The human palpebral fissure is the most elongated in all the primates and is believed to allow visual field expansion through ample eye movement^13^,^14^. We recently showed that, in humans, the median visual field surface area increased by 46% in the temporal area with eye abduction and that the horizontal median temporal eccentricity of the visual field was 94.7° in the primary position of gaze (up to 104.5°) and 128.3° with eye abduction (up to 137.7°)^7^. For the human orbit, the mathematical model used in the present report yields figures that are in the same range as these experimental results. Hence, in the primary position of gaze, it calculates a temporal visual field eccentricity (*ε max.*) of 103°. When the whole range of eye abduction is taken into account, it calculates a mean maximum temporal visual field eccentricity (*ω mean*) of 123.9°. For a 36.9° abduction, it calculates a top maximum temporal visual field eccentricity (*ω top max.*) of 136.1°.

Using computerized tomography, Saban *et al.* reported an 11.11% oculo-orbital index (OOI) in one *Hylobates lar*^15^. Photographs of live gibbons^16^,^17^ show that their eyeballs are deeply tucked into their orbits. However, in our model, the calculated OOI was 50.2% in the “Hylobatidae-like” orbit. There is therefore a considerable discrepancy between *in-vivo* facts about the eyeball position in Hylobatidae and the OOI calculated in the modified “Hylobatidae-like” human orbit. For this orbit, we considered our model’s relevance to be poor.

Schultz reported that, in chimpanzees, the eye lies deep within the orbit, thereby affording ample protection on all sides^18^. Furthermore, using computerized tomography, Saban *et al.* reported a 34.78% OOI in one *Pan troglodytes*^19^. Schultz noted that, in orangutans, the eyeball extends slightly beyond the orbital margin^18^, a fact confirmed by analysing live animal photographs^16^,^20^. Photographs of a live gorilla^16^,^20^,^21^,^22^ show that their eyeballs lie deep within their orbits, similar to chimpanzees. The calculated OOI was 42% in the “*Pan*-like” orbit, 29.6% in the “*Pongo*-like” orbit and 29.3% in the “*Gorilla*-like” orbit. We consider these values to be in accordance with the aforementioned *in-vivo* facts. We considered our model’s relevance to be good for these orbits. We therefore took into account the data yielded by our model for the “*Pan*-like”, “*Pongo*-like” and “*Gorilla*-like” modified human orbits. As the “*Pongo*-like” and “*Gorilla*-like” modified orbits have very similar *φ* values, we reported only the data pertaining to the “*Gorilla*-like” orbit in Figs 1 and 3.

Visual field (with or without permitted eye motion) cannot be tested in non-human apes. We designed the present study in an attempt to overcome this limitation. The basic idea behind this study was to use an archetypal human orbit, the opening angle (OA) of which can be varied so that it becomes “non-human ape-like”. Changing the orbit’s OA from 107.1° (human) to 98.7°, 94.3°, 94.4° and 101.6°, respectively, created “*Pan*-like”, “*Gorilla*-like”, “*Pongo*-like” and “Hylobatidae-like” modified human orbits. We have shown poor model relevance for Hylobatidae. We therefore considered that the figures generated by our model for Hylobatidae were unusable.

To some extent, the present study bridges the gap between anatomy and physiology. It shows that the rearward human lateral orbital margin offers much more eyeball abduction-related visual field expansion than that of non-human apes. More precisely, it shows that the 8.4° difference in OA (107.1°–98.7°) between human and *Pan* results in a 21.1° difference in maximum temporal visual field eccentricity (136.1°–115°) with permitted eye abduction. In other words, a minor 8.4° anatomical difference results in a large (2.5-fold) difference in maximum temporal visual field eccentricity. Similarly, it shows that the 12.8° difference in OA (107.1°–93.3°) between Human and *Gorilla* results in a 2.5-fold greater 31.8° difference in maximum temporal visual field eccentricity (136.1°–104.3°) with permitted eye abduction. The figures generated by our model for *Pongo* are very close to those obtained with *Gorilla*, the respective *φ* values of both genera being 94.3° and 94.4°. These figures are lower than the 94.9° neutral *φ* value. This implies that for the *Gorilla*-like and *Pongo*-like orbits, eye abduction is not “profitable” (visual-field efficient), so to speak, because it results in an average visual field with lower eccentricity than that recorded in the primary position of gaze.

In our model, for the human orbit, the *ω top max.* (and related *θ* and *α* angles values) and *ω mean* plot reached a plateau for the highest *φ* values. The plateau is the consequence of a maximum eyeball abduction of 45°.

The rearward human lateral orbital position could be a by-product of other aspects of craniofacial anatomy (an exaptation) as loss of the snout with facial retraction below the anterior cranial fossa^23^ or a steep forehead^24^. These two factors could have had opposite effects, the former being expected to drive the lower lateral orbital margin rearward and the latter being expected to drive the upper lateral orbital margin forward. Furthermore, compared with non-human apes, modern humans eat soft, highly processed foods and do not spend much time chewing^23^,^25^. Accordingly, modern humans have masticatory muscles that are much less developed than those of non-human apes^23^. In anthropoid primates, the line of action of the anterior temporalis muscle is roughly vertical^26^. In humans, posterior facial retraction has resulted in a more posteriorly placed anterior temporalis muscle, with a line of action which is expected to have more of an antero-posterior component than that observed in non-human apes. However, not much stress is transferred to the upper face, including the postorbital septum, during chewing, in anthropoid primates including humans^23^,^27^. Furthermore, assuming that there is more antero-posterior strain on the postorbital septum in humans than in non-human apes, the expected response would be to add bony mass^23^,^28^ to the zone under strain, the result of which would not be expected to change the position of the lateral orbital margin. Finally, the human anterior temporalis muscle, which is proportionally thinner than that in non-human apes, may provide less support for the postorbital septum. However, the influence of this factor on the position of the human rearward lateral orbital margin (RLOM) position is unsubstantiated. To quote Lieberman^29^: “heads defy many efforts to simplify because they are, by nature, complex and highly integrated systems”. Hence, the RLOM likely represents a compromise of many factors including the demands of temporal fossa content and those of the orbit. Apart from exaptation, there is good reason to believe that natural selection has driven the evolution of an RLOM in humans. The human RLOM does not offer much lateral eyeball protection, which may have had little negative selective pressure in humans. Indeed, humans live in a branchless environment with much less risk of branch-related eyeball trauma^4^ than the non-human apes who almost exclusively inhabit tropical forests^16^,^17^,^22^. Hence, far from being a “design fault” in the human visual system^30^, RLOM position and anterior eyeball position in the orbit may represent a trade-off between usually non-blinding UVB-related eyeball conditions (e.g. pterygia or cataracts of the nasal aspects of the crystalline lens^30^) and a large visual field^30^, enlarged through eye motion^7^,^12^,^13^, which may aid survival^30^. Humans are ground-dwellers^16^, live in open spaces more than in tropical forests^25^,^31^,^32^ and, being the only habitual mammalian bipeds^16^,^21^,^31^,^33^, have most of their visual targets at or parallel to ground level^4^. Compared to knuckle-walking, human bipedal locomotion involves a higher head position and a more forward-facing orbital plane orientation relative to the frontal plane^34^. This overlooking, forward-facing, orbital position is useful in humans whose large, heavy heads are much more difficult to move than those of smaller primates because the human head weight increases like the cube of the multiplier whereas the neck surface increases only like the square of the multiplier (Galileo’s principle of similitude^14^,^35^,^36^). In primates, the eye scales with greater negative allometry with respect to body mass than the orbit does^18^,^37^,^38^. The eyes of large primates (e.g. humans) therefore fill proportionally less orbital volume that the eyes of small primates^37^,^38^. Large primates therefore have proportionally more orbital space for oculomotor muscles^14^. Based on Galileo’s principle of similitude^14^,^35^,^36^, this favours swift and ample eyeball movements in large primates, especially humans^4^,^7^,^39^,^40^,^41^,^42^. Set in their overlooking anatomical position, human eyes may thus efficiently scan their environment, mostly at or parallel to ground level^4^. This process, which is very useful in challenging environments^12^, saves head movement and increases spatial awareness and vigilance through visual and visual field exploration, with the RLOM avoiding obstruction of the EMVF^4^,^5^,^6^,^7^.

The fact that the anatomical measurements of the OA in humans and non-human apes (denoted by *φ* in the present study) and human orbital width were recorded in the NOP enabled us to use previously published data by Cabanis *et al.* in 1033 normal adults aged 14 to 89 years^43^. Furthermore, using the NOP is a validated way of orienting the head in space to perform reliable inter-species comparisons^10^. The OA values used in this study come from a large sample of skulls^4^ (100 human skulls and 120 non-human ape skulls).

Our model has many limitations. It only assesses temporal visual field eccentricity in the NOP. It considers that the cornea is spherical whereas it is well know that the cornea is an aspherical diopter, the peripheral part of which is flatter than its apex^44^,^45^,^46^. It does not take into account the air-cornea then cornea-aqueous humor interface. Rather, it takes a single air-aqueous humor interface into account, considering that the cornea is infinitely thin. For that reason, as previously done before^47^,^48^, a 1.336 rather than a 1.376^49^,^50^ refractive index has been used for the cornea, as if the aqueous humor bulged forward and were in direct contact with the air.

Our model only takes into account rays refracted by the cornea through the pupil centre, whereas the pupil actually offers a wider area through which rays may be refracted. Our visual field calculations have not taken into account the difference between the optical axis and the visual axis. These axes are 5° apart in humans^47^,^48^,^51^, the area centralis being located slightly temporal to the visual axis. However, this approximation is acceptable in higher primates for which the two axes roughly coincide^52^. We have been unable to find data on the thickness of soft tissues in humans anterior to the temporal orbital margin in the NOP. The orbicularis oculi thickness anterior to the frontal process of the zygomatic bone was less than 1 mm on average in 40 healthy volunteers^53^. The average skin thickness in the lower eyelid (a zone close to the orbital margin) using full-thickness skin biopsies in 3 fresh cadavers was 0.82 mm^54^. We assigned a plain 1 mm thickness to soft tissues anterior to the temporal orbital margin in the NOP. This value seemed compatible with the rough estimate provided by the aforementioned data.

In our model, we considered that eyeball rotation in abduction was even around its center. In reality, this is probably more complex. Using magnetic resonance imaging, Lasudry *et al.* reported that for upgaze and downgaze, a translatory movement of the globe opposite to the direction of gaze occurred^55^,^56^. The same phenomenon could occur in horizontal eye movements.

Maximum eyeball excursion in humans and monkeys is similar, namely+/−45°^57^. Taking eyeball abduction into account in order to compare visual field eccentricity in humans and non-human apes therefore makes sense. In our model, for the “*Pan*-like” and “*Gorilla*-like” modified orbits, maximum temporal visual field eccentricity was reached at only 15.8° and 5.1°, respectively. Eccentricity decreased for higher abduction values.

To summarise, the human orbit differs from that of non-human apes in that its lateral orbital margin is significantly more rearward. This rearward position does not obstruct the lateral visual field, especially the additional visual field gained through eye motion. This additional visual field is therefore considered to be wider in humans than in non-human apes^4^. However, no attempt at quantifying this additional lateral visual field difference has ever been attempted. The mathematical model used in this study shows that the minor orbital anatomical differences between humans and non-human apes results in wide visual field expansion with eyeball abduction differences. More precisely, an 8.4° difference in the orbital margin position between humans and *Pan* leads to a 21.1° visual field expansion with the eyeball abduction difference (136.1° versus 115°). In a previous report, humans have been deemed to be unique among mammals in combining overlapping monocular visual fields and, through eye motion, large (enlarged) lateral visual fields^7^. The present report strongly suggests that such visual field characteristics make humans unique among hominoids.

## Methods

### Anatomical and physiological data

Table 2 sums-up the optical, physiological and anatomical data used in our mathematical model.

### Eyeball data

The cornea was considered as a spherical diopter^47^,^45^ with a 7.8-mm radius of curvature^44^,^49^ and a 12-mm horizontal diameter^58^. The air-cornea interface and the cornea-aqueous humor interface were combined into the air-cornea interface as before^47^,^48^. The cornea refractive index was set at 1.336^47^,^48^. A 24.19-mm antero-posterior eyeball length in the NOP was used^43^. The external ante-bicanthal eyeball segment in the NOP corresponded to the eyeball segment located anterior to the line joining the points where the NOP and both temporal orbital margins intersect. A value of 15.89 mm was used for this segment^43^. A 3.15-mm anterior chamber eyeball depth was used^49^. Eyeball rotation around the eyeball centre was employed. A 0° (primary position of gaze) to 45° eyeball abduction range was used^39^,^41^. A coinciding eyeball optic axis (axis of symmetry of the lens and cornea) and visual axis (the line through the centre of the corneal apex to the area centralis of the retina) were used^52^. Temporal rays refracting exclusively through the pupil centre (as opposed to the whole pupil area) were taken into account.

### Orbital data

**Figure 4:**
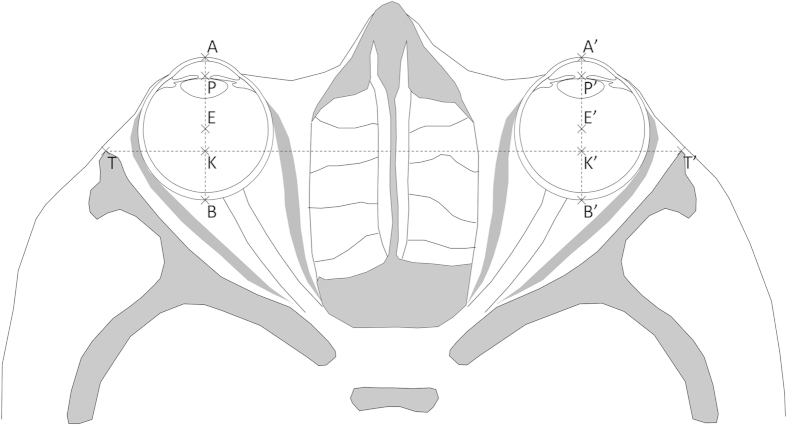
Schematic cross-section of human orbits in the neuro-ocular plane. A = cornea apex, P = pupil centre, E = eye centre, B = posterior pole of the eye, T = temporal orbital margin, K = intersection of AB and TT’; A’, P’, E’, B’, T’, K’ = symmetrical points on the other side. TT’ = 97.52 mm; PP’ = AA’ = EE’ = BB’ = 63.73 mm; AB = 24.19 mm; AK = 15.89 mm.

The neuro-ocular plane (NOP)^8^,^9^,^10^ was the reference plane. Many morphological data have been measured in this plane by Cabanis *et al.* using computerized tomography in 1033 normal adults aged 14 to 80 years^10^. Furthermore, this plane can be used for reproducible head orientation in space, making inter-species comparisons possible^10^,^59^. The NOP is defined as the plane which, in primary position of gaze (looking straight ahead in the distance), contains the centre of the crystalline lenses, optic discs, and optic foramina^8^,^9^,^10^. In the primary gaze position, the pupil is equidistant from the superior and inferior orbital margins^49^,^60^. We therefore defined the NOP, as Paul Broca did in 1873^60^, as the plane which runs symmetrically through both optic foramina and through a point located mid-way between the highest and lowest points of the orbital margin. The external bicanthal distance, that is the distance between the two points where the NOP and temporal orbital margins intersect (denoted by TT’ in Fig. 4), is 97.52 mm^43^. The inter-ocular distance, that is the distance between the centre of the two crystalline lenses (denoted by AA’ or PP’ or EE’ or KK’ or BB’ in Fig. 4), is 63.73 mm^43^. A 1-mm soft tissue thickness anterior to the temporal orbital margin was used.

### Orbital Opening Angle

**Figure 5:**
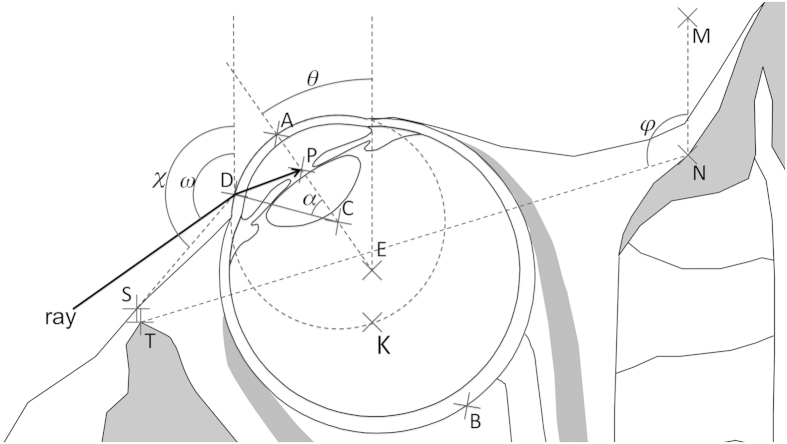
Schematic cross-section of the human orbit in the neuro-ocular plane. *φ* = opening angle ( = 107.1° in Human; 98.7° in *Pan*; 94.3° in *Gorilla*; 94.4° in *Pongo*; 101.6° in Hylobatidae), T = temporal orbital margin, N = nasal orbital margin, S = skin projection of T in a direction orthogonal to NT, A = cornea apex, P = pupil centre, C = cornea centre, E = eyeball centre, B = posterior pole of the eyeball, D = any point on the temporal part of cornea, ray = ray refracted at point D and passing through the pupil centre (P), *ω* = angle between “ray” and sagittal plane, *χ* = angle between SD and sagittal plane, *θ* = eyeball abduction angle (from 0° for primary position of gaze to 45° for maximum eye abduction). Any variation of angle *φ* modifies the position of points N and S. NS = 1 mm, NT = 40.17 mm.

The opening angle (OA) denotes the more or less rearward lateral orbital margin position. The higher this angle, the more rearward the lateral orbital margin position. Details about the OA measurement method used on 100 human skulls and 120 non-human ape skulls have already been published^4^. The average OA (+/−standard error of the mean) was 107.1° (+/−0.245°) in humans, 98.7° (+/−0.408°) in *Pan*, 94.3° (+/−0.401°) in *Gorilla*, 94.4° (+/−0.489°) in *Pongo* and 101.6° (+/−0.486°) in Hylobatidae^4^. The lowest OA value recorded was 85°, in the right orbit of a male *Gorilla beringei* and in the left orbit of a male *Pongo pygmaeus*^4^. The highest OA value was 115° in the left orbit of a male human from Romania^4^. In this study, the OA was denoted by *φ* (Fig. 5).

### Orbital diameter in the NOP

Thirty human skulls chosen at random (using labels placed in a ballot box and drawn at random) were used for the calculations: 6 skulls were from Europe (3 males, 3 females), 6 skulls were from Aboriginal Australians (3 males, 3 females), 6 skulls were from China (3 males, 3 females), 6 skulls were from Native Americans (1 male, 1 female, 4 unknown genera) and 6 skulls were from Africa (3 males, 3 females). In each skull, the NOP position was illustrated as described above. The orbital width of the NOP was measured on both sides using calipers (model “815A”, Facom, New Britain, CT, USA). The average (+/−standard error of the mean) of the sixty measurements was 40.17+/−0.277 mm.

### Mathematics

#### Principle of the mathematical model

**Figure 6:**
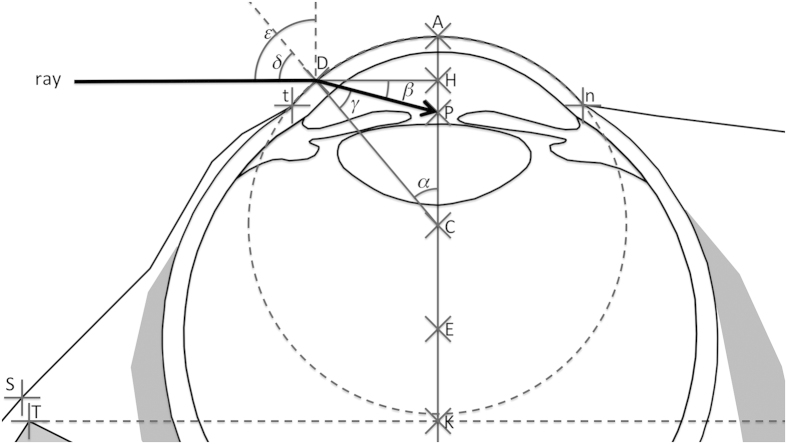
Schematic cross-section of the human eye in primary position of gaze in the neuro-ocular plane. A = cornea apex, D = any point on the temporal part of the cornea (position defined by angle *α* that varies between 0° and 50.3°), H = orthogonal projection of D on AC, P = pupil centre, C = cornea centre, E = eye centre, γ = angle PDC, β = angle HDP, ray = ray refracted at point D and passing through the pupil centre (P), *δ* = angle between “ray” and CD, *ε* = *δ* + *α* = angle between ray and sagittal plane, t = temporal edge of cornea, n = nasal edge of cornea, nt = 12 mm.

Our model involves points located in the NOP and displayed in Figs 4, 5 and 6. An overview of both orbits, one eyeball and one orbit are displayed in Figs 4, 5 and 6, respectively. Our approach consisted in using an archetypal human orbit based on solid published facts (see sections “Eyeball data”, “Orbital data”, “Orbital opening angle” and “Orbital diameter in the NOP”) and measured anatomical facts in 100 skulls from 5 continents^4^. We then modifying one and only one parameter, i.e. the lateral orbital margin position of the archetypal human orbit. More precisely, by allocating the archetypal human orbit the lateral orbital margin position measured in Hylobatidae (30 skulls), *Pan* (30 skulls), *Pongo* (30 skulls) and *Gorilla* (30 skulls), we created Hylobatidae-like, *Pan*-like, *Pongo*-like and *Gorilla*-like human orbits. Thus, the model involves mathematical calculations on the archetypal human orbit and on modified human orbits using variations of the OA (denoted by *φ*: see Fig. 5). For instance, by modifying the human *φ* from 107.1° (archetypal human orbit) to 98.7° or 94.3° (values measured in *Pan* and *Gorilla*, respectively), we created modified “*Pan*-like” or “*Gorilla*-like” human orbits. We would like to make it clear that a *Pan*-like or a *Gorilla*-like human orbit is not the same as a *Pan* orbit or a *Gorilla* orbit.

#### Terminology used (Figure 6)

The angle between any given temporal ray refracted through the pupil centre and the sagittal plane, with the eye in the primary position of gaze, was called *ε*. The angle between the sagittal plane and the most peripheral ray refracted through the pupil centre, with the eye in the primary position of gaze, was called *ε max.* The angle between the sagittal plane and any temporal ray refracted through the pupil centre, with the eye in abduction (denoted by *θ*), was called *ω*. The angle between the sagittal plane and the most peripheral ray refracted through the pupil centre, with the eye in one given *θ* abduction position, was called *ω max.* The angle between the sagittal plane and the most peripheral ray refracted through the pupil centre for the whole range of *θ* values (between 0° and 45° abduction) was called *ω top max.* The mean value of all *ω max.* values (for *θ* values between 0° and 45°) was called *ω mean*.

#### Temporal visual field in primary position of gaze: ε angle computation

The points and angles referred to are displayed in Figs 4, 5 and 6.

The *ε* angle was computed using the following equations:


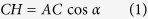



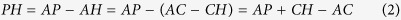



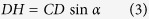



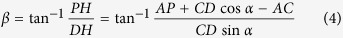



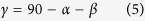


The air and cornea refractive indices are 1 and 1.336, respectively. Then, according to Snell-Descartes law:


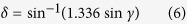


And then:


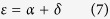


#### Points K, S, and N computation

Let us consider the orthonormal basis 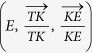



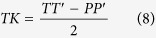



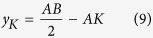


*φ*_*h*_ refers to the value of *φ* in humans.

For the archetypal human orbit, with the angle values given in degrees, the point S coordinates are:


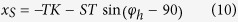



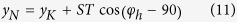


The point N coordinates are then:


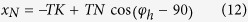



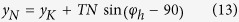


Let us now consider any *φ* value. For any modified human orbit (in which *φ* ≠ *φ*_*h*_), with the angle values given in degrees, given that


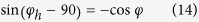



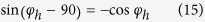



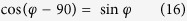


and that


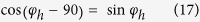


then the point S coordinates are:


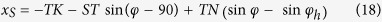



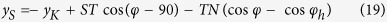


#### Temporal visual field with eye abduction: ω angle computation


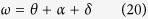


for *θ* = 0(primary position of gaze), *ω *= *ε*

#### Angle *χ* (critical angle) computation

Point C coordinates are:


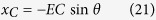



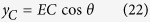


Point D coordinates are:


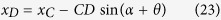



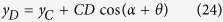


Then:


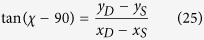


and:


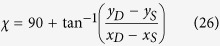


#### Computation of maximum visual field angles (ε max., ω max., ω top max.) and of ω mean

Angle *χ* is the critical angle: the ray is refracted through the pupil center if *ω* < *χ* (or, for *θ* = 0, if *ε* < *χ*). Using the aforementioned equation, a computer program was written to calculate the values of *α* and *θ* yielding the *ω max.* value for a given *φ* value.

### For a given *φ* angle value

The *ε max.* value was computed by setting the *θ* value to zero and varying the *α* values only. The *ω max.* was computed by setting the *θ* angle value and varying the *α* angle values using 0.001 degree increments. Using dichotomy with 0.001 degree increments for the *θ* and *α* angles values, the comprehensive set of *ω max.* values was computed. The *ω top max.* value was computed by selecting the highest set value. The *ω mean* value was computed by averaging the whole set values.

### Neutral *φ* angle value computation

The neutral *φ* value, i.e. the *φ* value that results in equal *ω mean* and *ε max.* values taking the whole range of eye abduction (*θ* from 0 to 45°) into account, was computed using dichotomy.

#### Oculo-orbital indices for modified (“non-human ape-like”) human orbits

In the NOP, the oculo-orbital index (OOI) denotes the proportion of the eyeball located anterior to the lateral bicanthal line (TT’ line in Fig. 4). The OOI is 65.7% in humans^43^. Using our coordinates system (Figs 4, 5 and 6):


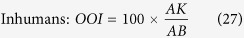


In non-human ape-like orbits, with *φ*_*h*_ and *φ* expressed in degrees:


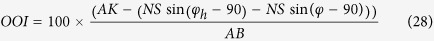


The OOIs were calculated in non-human apes to compare the data yielded (predicted) by our mathematical model to published data or published *in-vivo* facts regarding the more or less anterior eyeball position into the orbit. In doing so, we aimed to evaluate the relevance of our mathematical model in order to discuss results only in accordance with published data.

## Additional Information

**How to cite this article**: Denion, E. *et al.* Human rather than ape-like orbital morphology allows much greater lateral visual field expansion with eye abduction. *Sci. Rep.* **5**, 12437; doi: 10.1038/srep12437 (2015).

## Figures and Tables

**Table 1:** Temporal visual field angles in primary position of gaze and in eye abduction for the human orbit and non-human ape-like modified orbits.

		Hylobatidae-like orbit	*Gorilla*-like orbit	*Pongo*-like orbit	*Pan*-like orbit	Human orbit	“Neutral” orbit^a^	Lowest opening angle	Highest opening angle
Opening angle (*φ*)		101.6° ± 0.486°	94.3° ± 0.401°	94.4° ± 0.489°	98.7° ± 0.408°	107.1° ± 0.245°	94.9°	85°^b^	115°^c^
	Skulls studied	30	30	30	30	100	NA	2	1
ε max		103°	103°	103°	103°	103°	103°	82.6°	103°
	Related α	50.3°	50.3°	50.3°	50.3°	50.3°	50.3°	34.8°	50.3°
ω top max.		122.2°	104.3°	104.5°	115°	136.1°	105.6°	82.6°	148°
	Related α	46.9°	46.9°	46.9°	46.9°	46.9°	46.9°	34.8°	50.3°
	Related θ	23°	5.1°	5.3°	15.8°	36.9°	6.4°	0°	45°
ω mean		117.6°	101.4°	101.7°	112.2°	123.9°	103°	75.3°	125.5°

The opening angle (*φ*) values are expressed as the mean +/−standard error of the mean (in brackets).

^a^The neutral orbit has a *φ* angle resulting in an *ω mean* value that is equal to that of *ε max.* taking the whole range of *θ* values (between 0 and 45°) into account.

^b^The lowest *φ* recorded value was 85°, in the right orbit of a male *Gorilla beringei* and in the left orbit of a male *Pongo pygmaeus*.

^c^The highest *φ* value was 115° in the left orbit of a male human from Romania.

**Table 2:** Optical, physiological and anatomical data used in our physiological model.

Optical, physiological and anatomical data	Hypotheses
Corneal shape	Spherical
Corneal thickness	None^a^
Corneal radius of curvature	7.8 mm
Horizontal corneal diameter	12 mm
Air refractive index	1
Corneal refractive index	1.336^b^
Eyeball axial (antero-posterior) length	24.19 mm
External ante bi-canthal eyeball segment in the NOP	15.89 mm
Anterior chamber depth	3.15 mm
Eyeball rotation	Regular, around its centre
Eyeball abduction range	0 to 45°
Eyeball visual axis	Coincides with optic axis
Refraction of temporal rays by the cornea	Through pupil centre exclusively
Soft tissues thickness anterior to the temporal orbital rim	1 mm
Bi-canthal external distance	97.52 mm
Inter-ocular distance	63.73 mm
Orbital width in the NOP	40.17 mm
Average orbital opening angle value (human)	107.1°
Average orbital opening angle value (Pan)	98.7°
Average orbital opening angle value (Gorilla)	94.3°
Average orbital opening angle value (Pongo)	94.4°
Average orbital opening angle value (Hylobatidae)	101.6°
Lowest orbital opening angle value	85°
Highest orbital opening angle value	115°

Abbreviation used: NOP = neuro-ocular plane.

Notes:

^a^in our model the air-cornea interface and the cornea-aqueous humor interface are combined into the air-aqueous humor interface, as if the cornea was infinitely thin and the aqueous humor bulged forward and was in direct in contact with the air.

^b^According to point “a”, the cornea refractive index was set at 1.336 (and not 1.376), like aqueous humor.
