# Corrigendum: Human rather than ape-like orbital morphology allows much greater lateral visual field expansion with eye abduction

**DOI:** 10.1038/srep19389

**Published:** 2016-01-20

**Authors:** Eric Denion, Martin Hitier, Eric Levieil, Frédéric Mouriaux

Scientific Reports 5: Article number: 12437; 10.1038/srep12437 published online: 07202015; updated: 01202016.

This Article contains errors in Reference 40 which was incorrectly given as ‘Le Grand, Y. *Chapitre 13: L’orbite et ses muscles*. In *Optique Physiologique. Tome Troisième: l’Espace Visuel* (ed. Le Grand, Y.) 28–41 (Editions de la “Revue d’Optique”, Paris, 1956)’. The correct reference is listed below:

Le Grand, Y. *Chapitre 13: L’orbite et ses* muscles. In Optique Physiologique. *Tome premier : la dioptrique de l’œil et sa correction.* (ed. Le Grand, Y.) 153–163 (Editions de la “Revue d’Optique”, Paris, 1956).

